# Clinical predictors of mortality in immune thrombotic thrombocytopenic purpura: A National Inpatient Sample analysis

**DOI:** 10.1111/bjh.70686

**Published:** 2026-07-12

**Authors:** Anand Shah, Christopher Chen, Ilana Pyatetsky, Shruti Chaturvedi, Ankit S. Shah

**Affiliations:** ^1^ Department of Medicine Rutgers New Jersey Medical School Newark New Jersey USA; ^2^ Division of Hematology, Department of Medicine Johns Hopkins University School of Medicine Baltimore Maryland USA; ^3^ Division of Hematology/Oncology Department of Medicine Rutgers New Jersey Medical School Newark NJ USA; ^4^ Rutgers Cancer Institute Newark NJ USA

**Keywords:** acute kidney injury, in‐hospital mortality, National Inpatient Sample, sepsis, therapeutic plasma exchange, thrombotic thrombocytopenic purpura

To the Editor,

Thrombotic thrombocytopenic purpura (TTP) is a rare but life‐threatening thrombotic microangiopathy (TMA) caused by severe ADAMTS13 deficiency, leading to platelet‐rich microvascular thrombosis, thrombocytopenia, microangiopathic haemolytic anaemia and ischemic organ injury.^1^, ^2^ Before the advent of therapeutic plasma exchange (TPE), mortality exceeded 90%, but outcomes improved substantially with TPE and immunosuppressive therapy.^3^, ^4^ More recently, caplacizumab has changed the management of immune‐mediated TTP (iTTP) by reducing refractoriness and time to platelet recovery.^5^, ^6^ International guidelines recommend immediate initiation of TPE with corticosteroids and caplacizumab for patients with suspected iTTP.^7^ Despite these advances, national data describing predictors of in‐hospital mortality remain limited. We used the National Inpatient Sample (NIS) from 2016 to 2022 to evaluate clinical predictors of in‐hospital mortality among adult hospitalizations with suspected iTTP, with particular focus on the timing of TPE.

We conducted a retrospective study using the Healthcare Cost and Utilization Project NIS from 2016 to 2022.^8^ Adult hospitalizations were included if they had a diagnosis code for thrombotic microangiopathy (ICD‐10‐CM M31.1) and a procedure code for TPE during the same admission, consistent with previously used administrative database definitions.^9^, ^10^ Hospitalizations with diagnostic codes for haemolytic uremic syndrome or procedure codes for eculizumab administration were excluded to reduce misclassification with non‐TTP TMAs. Each discharge was treated as an independent hospitalization. The primary exposure was the timing of TPE, categorized as early (≤1 day from admission) or delayed (>1 day) and the primary outcome was in‐hospital mortality. Covariates included age, sex, race/ethnicity, selected comorbidities, triggering conditions and in‐hospital complications identified using ICD‐10‐CM/PCS codes.

All analyses incorporated NIS survey design variables and discharge weights. Survey‐weighted logistic regression was used to identify predictors of in‐hospital mortality. Model A included demographic and baseline clinical variables, while Model B additionally included in‐hospital complications. Sensitivity analyses included a simpler model adjusted only for age, sex and race/ethnicity and a model excluding hospitalizations with pregnancy, coronavirus disease 2019 (COVID‐19) infection, malignancy, transplant history or transplant procedure during the index hospitalization. ICD‐10‐CM/PCS codes are provided in Table S1.

A total of 12,095 nationally weighted hospitalizations meeting the study definition for TTP were identified. Median age was 50 years (interquartile range [IQR], 36–62), 66.8% were female and 42.5% were Black. Annual prevalence remained relatively stable, ranging from 5.2 to 6 per 100 000 hospitalizations. Overall in‐hospital mortality was 8.8% and fluctuated without a clear temporal trend during 2016–2022, ranging from 6.9% to 10.1% (Figure 1). Non‐survivors were older than survivors, more frequently White and more likely to have Medicare insurance and higher total hospital charges (Table S2). Mortality was lower among hospitalizations with early TPE than delayed TPE (5.6% vs. 14.0%, *p* < 0.001). Early TPE was associated with shorter length of stay and lower total hospital charges (Figure S1).

**FIGURE 1:**
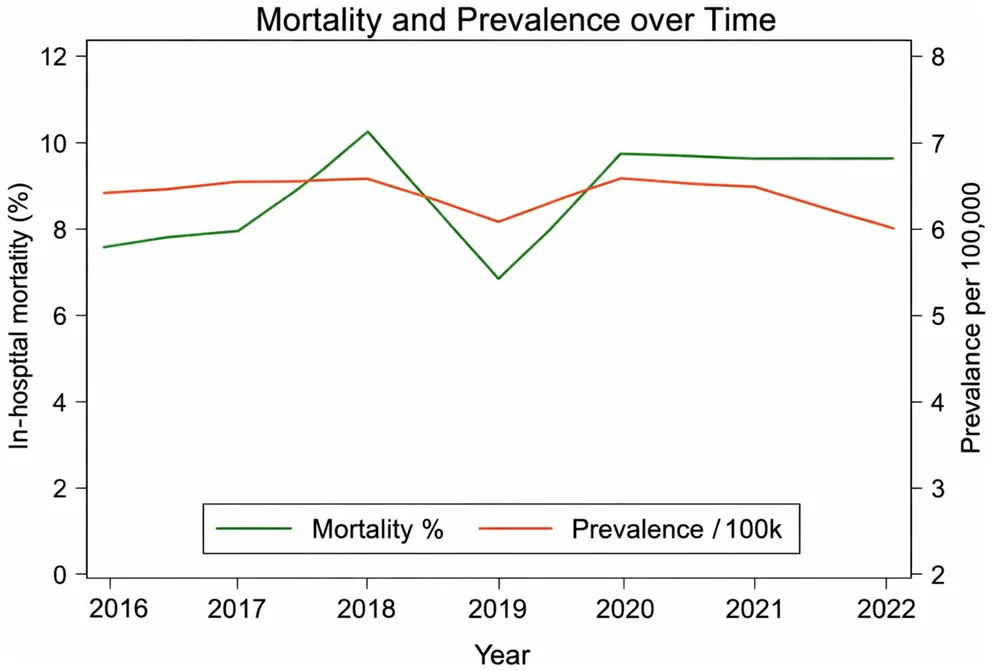
Trends in the prevalence and in‐hospital mortality of thrombotic thrombocytopenic purpura, 2016–2022.

Non‐survivors had a higher comorbidity burden than survivors and were more likely to have chronic kidney disease, chronic lung disease, cirrhosis, chronic heart failure, COVID‐19, malignancy and solid organ transplant history (Table S3). Major in‐hospital complications were also more common among non‐survivors, including sepsis, acute kidney injury (AKI), gastrointestinal bleeding, acute heart failure, cardiac arrest, neurological complications, mechanical ventilation and vasopressor use (Table S4).

In multivariable analysis adjusting for demographic and baseline clinical factors, early TPE remained independently associated with lower in‐hospital mortality (adjusted odds ratio [aOR] 0.49, 95% confidence interval [CI] 0.36–0.68). Older age, chronic lung disease, chronic heart failure, COVID‐19 and solid or haematological malignancy were associated with higher mortality, while Black race was associated with lower adjusted mortality compared with White race. When in‐hospital complications were incorporated, early TPE remained associated with lower mortality (aOR 0.66, 95% CI 0.47–0.92). Sepsis, acute kidney injury, acute heart failure, neurological complications and gastrointestinal bleeding were independently associated with higher mortality, while acute myocardial infarction was not independently associated with mortality (Figure 2; Table S5). When the timing of TPE was modelled continuously, each additional day to TPE initiation was associated with a 6% increase in adjusted odds of in‐hospital mortality.

**FIGURE 2:**
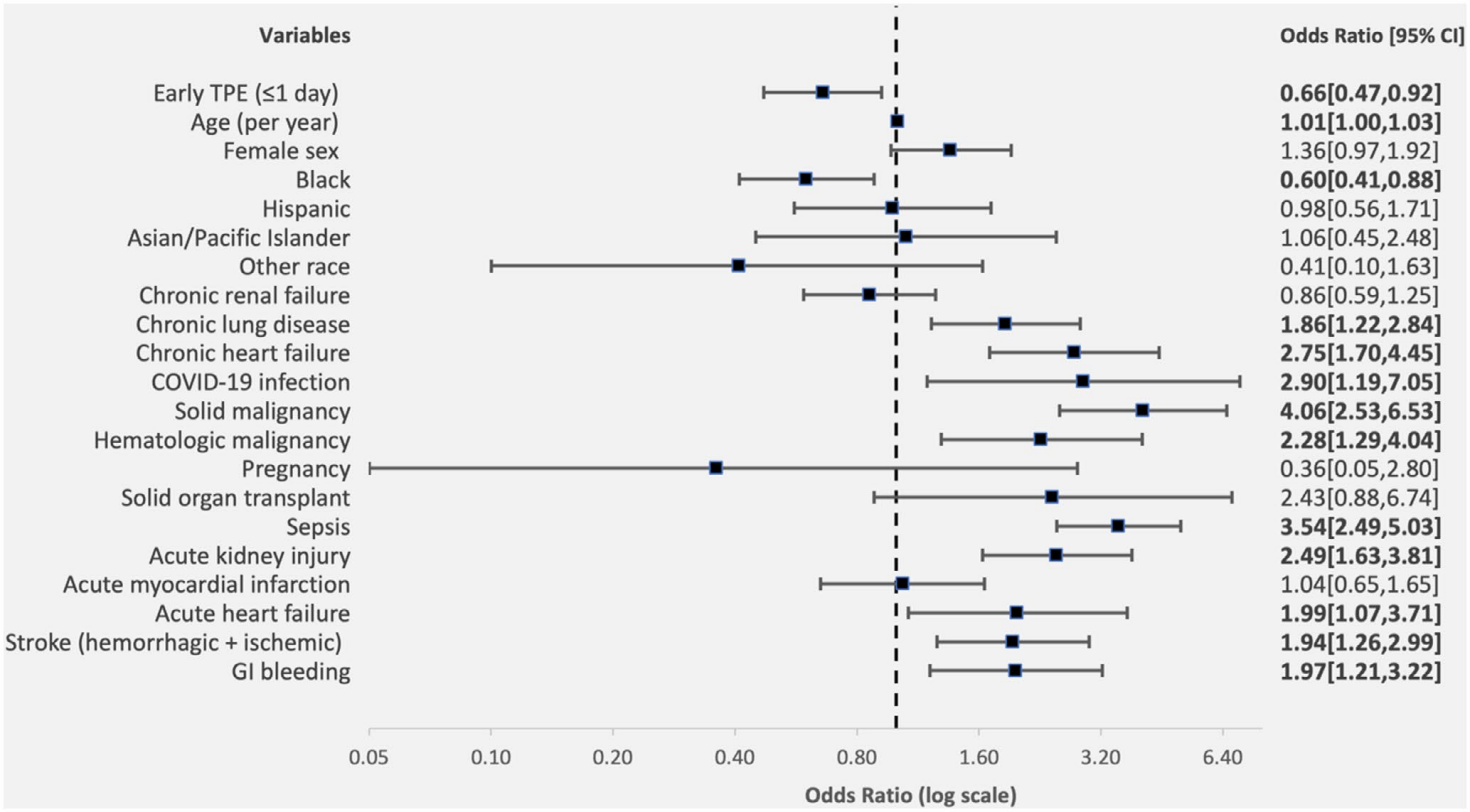
Forest plot of adjusted predictors of in‐hospital mortality in thrombotic thrombocytopenic purpura. Adjusted odds ratios with 95% confidence intervals are shown from the survey‐weighted multivariable model incorporating demographic variables, baseline clinical factors and in‐hospital complications. The dashed line denotes odds ratio = 1.

The association between early TPE and lower mortality remained consistent in sensitivity analyses. After excluding hospitalizations with pregnancy, COVID‐19, malignancy, transplant history or transplant procedure during the index hospitalization, early TPE remained associated with lower mortality in the baseline clinical model (aOR 0.50, 95% CI 0.34–0.73) and after incorporation of in‐hospital complications (aOR 0.65, 95% CI 0.44–0.97) (Table S6). In a simpler model adjusted only for age, sex and race/ethnicity, early TPE remained associated with lower mortality (aOR 0.42, 95% CI 0.31–0.57) (Table S7).

In this large national cohort, early TPE was one of the strongest modifiable predictors of survival among TTP hospitalizations. This association remained significant after adjustment for in‐hospital complications and across sensitivity analyses addressing possible secondary TMA misclassification. These findings reinforce that TTP has a narrow therapeutic window and that rapid recognition and early treatment remain critical to survival.

In our cohort, in‐hospital mortality was 8.8% with no clear temporal trend from 2016 to 2022, consistent with prior national estimates reporting 7.5%–11.1% over the preceding decade, suggesting a persistent mortality plateau despite therapeutic advances.^10^, ^11^ Although caplacizumab has demonstrated improved survival in large real‐world cohorts, including a 3‐month survival benefit in the Capla 1000+ study, its effect could not be evaluated in our analysis because medication use is not reliably captured in the NIS.^6^ Only 10–12 patients in our cohort were coded as receiving caplacizumab, likely reflecting coding inconsistency and limited population‐level uptake, potentially related to cost and variable access outside specialty centres. COVID‐19 was independently associated with mortality in our analysis (aOR 2.90); however, sensitivity analyses excluding COVID‐19 and other secondary TMA contexts did not materially alter the primary findings. These observations suggest that persistent mortality reflects system‐level barriers, including delays in diagnosis, delayed TPE initiation and inconsistent uptake of guideline‐recommended adjunctive therapies, rather than therapeutic inadequacy alone.

Early TPE was strongly associated with survival. Each day of delay increased odds of death by 6%, while initiation within 1 day halved mortality risk. After adjusting for in‐hospital complications, this protective association was partially attenuated, suggesting that early TPE may primarily prevent downstream organ failure rather than reverse established injury. This is consistent with prior data showing that delayed TPE is associated with clinical deterioration and early death.^12^ These findings support current practice paradigms that suspected iTTP should be treated urgently; even before confirmatory ADAMTS13 results are available.

Sepsis and AKI emerged as the strongest complication‐related predictors of mortality, likely functioning as markers of evolving multiorgan failure in severe TTP. Once established, sepsis and AKI may reduce the reversibility of organ injury even with prompt TPE. Acute heart failure, rather than acute myocardial infarction, independently predicted mortality. This pattern is consistent with the known cardiac involvement in TTP, in which myocardial injury reflects diffuse microvascular thrombosis rather than classic obstructive coronary disease.^13^ Neurological complications, including stroke, were independently associated with mortality, consistent with findings from the French and United States Thrombotic Microangiopathy (USTMA) registries linking neurological involvement to early death and refractoriness.^2^, ^13^ gastrointestinal bleeding, although uncommon, also independently predicted mortality, likely reflecting advanced systemic involvement and severe disease.

Among baseline clinical factors, chronic heart failure, chronic lung disease, COVID‐19 and malignancy were associated with higher mortality. These associations may reflect diminished physiological reserve, systemic inflammation or endothelial injury. Notably, the TMA plus TPE case definition used in this and prior NIS studies may capture some non‐TTP TMAs, particularly cancer‐associated or COVID‐19‐associated TMA that can receive empiric TPE before diagnostic confirmation, an inherent limitation of administrative database studies of TTP. In the sensitivity analysis excluding hospitalizations with pregnancy, COVID‐19, malignancy and transplant, early TPE remained associated with lower mortality, though some comorbidity and complication associations were attenuated, likely reflecting reduced sample size and removal of high‐acuity subgroups. Autoimmune conditions, despite being well‐recognized triggers of iTTP, were not associated with increased mortality, consistent with emerging evidence that autoimmune‐associated TTP may have comparable outcomes to primary iTTP when treated promptly.^14^ Black patients accounted for the largest racial group in this cohort and had lower adjusted mortality than White patients, consistent with prior studies.^15^ However, the mechanism remains unclear, and this finding should be considered hypothesis‐generating.

This analysis benefits from the size and national scope of the NIS, providing a broad view of TTP hospitalizations across different hospitals and patient populations. Several limitations should be noted. Case identification depends on ICD‐10‐CM/PCS coding and ADAMTS13 activity is not available. The database lacks laboratory values, platelet response, neurological findings, disease severity, relapse, outpatient deaths and specific treatments, including corticosteroids, rituximab and caplacizumab. TPE timing is based on procedure‐day coding and may not fully capture true treatment initiation, particularly for transferred patients or those treated before admission. Finally, because the NIS records hospitalizations rather than individual patients, recurrent TTP episodes may appear as separate encounters.

In conclusion, early initiation of TPE was independently associated with lower in‐hospital mortality among TTP hospitalizations in a contemporary national cohort. These findings reinforce current practice paradigms of early empiric treatment in suspected iTTP and highlight the importance of system‐level strategies to minimize delays in plasma exchange initiation.

## AUTHOR CONTRIBUTIONS

**Shruti Chaturvedi:** Writing – review and editing; validation. **Christopher Chen:** Writing – review and editing; writing – original draft; conceptualization. **Ankit S. Shah:** Conceptualization; methodology; project administration; supervision. **Anand Shah:** Conceptualization; methodology; formal analysis; project administration; writing – review and editing; writing – original draft. **Ilana Pyatetsky:** Writing – original draft; writing – review and editing.

## FUNDING INFORMATION

No specific funding was received for this study.

## CONFLICT OF INTEREST STATEMENT

The authors declare no conflicts of interest relevant to this work.

## ETHICS STATEMENT

This study used de‐identified, publicly available administrative data and was exempt from institutional review board review.

## PATIENT CONSENT STATEMENT

Patient consent was not required because the study used de‐identified administrative data.

## Supporting information


**Figure S1.** Length of stay and total hospital charges stratified by early versus late initiation of plasma exchange.
**Table S1.** ICD‐10‐CM/PCS codes used for variable definitions.
**Table S2.** Baseline characteristics of the study cohort and comparison between survivors and non‐survivors.
**Table S3.** Comorbidities and potential triggering conditions stratified by in‐hospital survival.
**Table S4.** In‐hospital complications stratified by survival status.
**Table S5.** Multivariable survey‐weighted logistic regression for predictors of in‐hospital mortality in TTP (Model A + B).
**Table S6.** Sensitivity analysis excluding hospitalizations with pregnancy, COVID‐19, malignancy, transplant history or transplant procedure during the index hospitalization.
**Table S7.** Sensitivity analysis using a demographic adjustment model.

## Data Availability

The data underlying this study are publicly available from the Healthcare Cost and Utilization Project National Inpatient Sample, subject to HCUP data use agreements.
