# Supplementary material for: Clinical predictors of mortality in immune thrombotic thrombocytopenic purpura: A National Inpatient Sample analysis
Source: Br J Haematol. 2026 Jul 12;209(3):1415–8. doi: 10.1111/bjh.70686 (PMC13570204; doi:10.1111/bjh.70686)
Supplement: Supplementary file 1 [file BJH-209-1415-s001.docx]

**Supplementary Table S1. ICD-10-CM/PCS Codes Used for Variable Definitions.**

| **Variable** | **ICD-10 Codes (Formatted)** |
| --- | --- |
| TTP | M31.1 |
| Therapeutic plasma exchange | 6A550Z3, 6A551Z3 |
| Eculizumab administration | XW043C6, XW033C6 |
| Hemolytic uremic syndrome | D59.32, D59.39 |
| Hemodialysis | 5A1D00Z–5A1D90Z |
| Sepsis | A40.x, A41.x, R65.2x, A02.1 |
| Chronic kidney disease | N18.x |
| Diabetes mellitus | E10.x–E14.x |
| Hypertension | I10.x, I15.x, I1A.x |
| Obesity | E66.x, Z68.3–Z68.4 |
| Chronic obstructive pulmonary disease | J41.x–J44.x |
| Asthma | J45.x |
| HIV | B20.x, Z21 |
| Cirrhosis / chronic liver disease | K70.x, K71.7, K73.x, K74.x, K75.4, K75.8 |
| Solid organ transplant (history) | Z94.0–Z94.9 |
| Hematopoietic stem cell transplant (history) | Z94.81, Z94.84 |
| Mechanical ventilation | 5A09.x, 5A19.x |
| Vasopressor use | 3E033XZ, 3E043XZ, 3E030XZ, 3E040XZ |
| Bacteremia | R78.81 |
| COVID-19 | U07.1, U00.x, U49.x |
| Coronary artery bypass grafting | 0210.x–0213.x |
| Acute pancreatitis | K85.x |
| Systemic lupus erythematosus | M32.x |
| Graves disease | E05.0 |
| Immune thrombocytopenia | D69.3 |
| Addison disease | E27.1, E27.2 |
| Sjögren syndrome | M35.0 |
| Scleroderma | M34.x |
| Rheumatoid arthritis | M05.x, M06.x |
| Vitiligo | L80 |
| Acquired factor VIII inhibitor | D68.311, D68.4, D68.04 |
| Malignancy | C00.x–C96.x |
| Acute kidney injury | N17.x |
| Acute myocardial infarction | I21.x–I24.x, I25.6 |
| Coronary artery disease | I20.x, I25.x |
| Acute heart failure | I50.21, I50.23, I50.31, I50.33, I50.41, I50.43, I50.811–I50.814 |
| Chronic heart failure | I50.1x–I50.9 |
| Paroxysmal tachycardia | I47.x |
| Atrial fibrillation / flutter | I48.x |
| Ventricular fibrillation / flutter | I49.0 |
| Cardiac arrest | I46.x |
| Cardiogenic shock | R57.0 |
| Venous thromboembolism (DVT/PE) | I26.x, I82.x |
| Coma | R40.2 |
| Seizure | G40.x |
| Ischemic stroke | I63.x |
| Intracranial hemorrhage | I60.x–I62.x |
| History of cerebrovascular disease | Z86.73, I69.x |
| Thrombocytopenia | D69.5, D69.6 |
| Transfusion-related acute lung injury (TRALI) | J95.84 |
| Platelet transfusion | 30233R0–30233R1, 30243R0–30243R1 |
| Allogenic Hematopoietic stem cell transplant | **30233G2, 30233G3, 30233U2–30233U4, 30233X2–30233X4, 30233Y2–30233Y4, 30243G2–30243G4, 30243U2–30243U4, 30243X2–30243X4, 30243Y0, 30243Y2–30243Y4** |

Abbreviations: TTP = thrombotic thrombocytopenic purpura; HIV = Human Immunodeficiency Virus; COVID-19 = coronavirus disease of 2019

**Supplementary Table S2: Baseline Characteristics of the Study Cohort and Comparison between Survivors and Non-Survivors.**

*(All percentages are weighted national estimates using HCUP discharge weights.  Denominators vary across variables due to missingness (see footnotes).*

|  | **Variable** | **Entire Cohort (Weighted n, % or Median [IQR])** | **Survivors (Weighted n, % or Median [IQR])** | **Non-Survivors (Weighted n, % or Median [IQR])** | **p-value** |
| --- | --- | --- | --- | --- | --- |
| **Demographics** | Age, years - median | 50 (36-62) | 48.5 (35-62) | 62 (48-70) | **<0.001** |
|  | **Age group** |  |  |  | **<0.001** |
|  | Age <60 years | 8,320 (68.8%) | 7840 (71%) | 480 (45%) |  |
|  | Age ≥60 years | 3,775 (31.2%) | 3190 (29%) | 580 (55%) |  |
|  | **Sex** |  |  |  | 0.30 |
|  | Male | 4015 (33.20%) | 3625 (33%) | 385 (36%) |  |
|  | Female | 8080 (66.80%) | 7405 (67%) | 675 (64%) |  |
|  | **Race/Ethnicity *** |  |  |  | **<0.001** |
|  | White | 4700 (40.12%) | 4085 (38%) | 615 (60%) |  |
|  | Black | 4980 (42.51%) | 4705 (44%) | 270 (26%) |  |
|  | Hispanic | 1330 (11.35%) | 1225 (11%) | 105 (10%) |  |
|  | Other | 705 (6.02%) | 655 (6%) | 50 (5%) |  |
| **Socioeconomics** | **Insurance** |  |  |  | **<0.001** |
|  | Medicare | 3550 (29.39%) | 3105 (28%) | 445 (42%) |  |
|  | Medicaid | 2800 (23.18%) | 2610 (24%) | 190 (18%) |  |
|  | Private | 4645 (38.45%) | 4270 (39%) | 375 (35%) |  |
|  | Self-pay | 685 (5.67%) | 655 (6%) | 30 (3%) |  |
|  | Other | 400 (3.31%) | 375 (3%) | 20 (2%) |  |
|  | **Income quartile (%)** |  |  |  | **0.03** |
|  | 0-25th percentile | 4085 (34.43%) | 3765 (35%) | 315 (30%) |  |
|  | 26th to 50th percentile | 2920 (24.61%) | 2710 (25%) | 210 (20%) |  |
|  | 51st to 75th percentile | 2750 (23.18%) | 2435 (23%) | 315 (30%) |  |
|  | 76th to 100th percentile | 2110 (17.78%) | 1900 (18%) | 210 (20%) |  |
| **Hospital Factors** | **Region** |  |  |  | 0.29 |
|  | Northeast | 2255 (18.64%) | 2035 (18%) | 220 (21%) |  |
|  | Midwest | 2700 (22.32%) | 2430 (22%) | 270 (25%) |  |
|  | South | 5290 (43.74%) | 4885 (44%) | 400 (38%) |  |
|  | West | 1850 (15.30%) | 1680 (15%) | 170 (16%) |  |
|  | **Teaching status** |  |  |  | 0.08 |
|  | Rural | 100 (0.83%) | 80 (0.7% | 20 (2%) |  |
|  | Urban nonteaching | 1200 (9.92%) | 1065 (10%) | 135 (13%) |  |
|  | Urban teaching | 10795 (89.25%) | 9885 (89%) | 905 (85%) |  |
| **Admission** | **Admission day** |  |  |  | 0.31 |
|  | Weekday | 9455 (78.17%) | 8655 (78%) | 800 (75%) |  |
|  | Weekend | 2640 (21.83%) | 2375 (21%) | 260 (24%) |  |
| **Utilization** | LOS (median) | 11 (IQR 7 – 18) | 11 (7-18) | 11 (6-22) | 0.55 |
|  | Total charges ($) | 178,789.5 (IQR 100,703 – 342,266) | 175,494 (99,554-332,011) | 220,257 (110,416-513,520) | **<0.001** |
|  | Time to Plasmapheresis, days (median [IQR]) | 1 (0-3) | 1 (0-3) | 2 (1-8) | **<0.001** |

*Data represent weighted national estimates. Continuous variables are presented as medians (IQR) or means (95% CI). Categorical comparisons were performed using survey-adjusted chi-square tests; continuous variables were compared using survey-weighted linear regression.*

*Race/ethnicity categories are based on the HCUP NIS uniform race variable, in which Hispanic ethnicity takes precedence over race. Thus, “White” and “Black” represent non-Hispanic individuals.* *Race “Other” includes Asian/Pacific Islander, Native American, and Other.*

*^ Non-routine includes transfer to short-term hospital, SNF/ICF/other facility, home health care, and discharge against medical advice.*

**Supplementary Table S3: Comorbidities and Potential Triggering Conditions Stratified by In-Hospital Survival**

| **Variable** | **Survivors (Weighted N, %)** | **Non-survivors (Weighted N, %)** | **p - value** |
| --- | --- | --- | --- |
| **Comorbidity burden** |  |  |  |
| Elixhauser comorbidity sum, mean (95% CI) | 4.49 (4.40-4.59) | 6.25 (5.97-6.54) | **<0.001** |
| Hypertension | 6,645 (60.2%) | 660 (62.3%) | 0.57 |
| Diabetes Mellitus | 2,170 (19.7%) | 235 (22.2%) | 0.39 |
| Chronic Kidney Disease | 2,450 (22.2%) | 345 (32.5%) | **<0.001** |
| Chronic Pulmonary disease (COPD + Asthma) | 1,445 (13.1%) | 250 (23.6%) | **<0.001** |
| HIV | 260 (2.4%) | 24 (2.4%) | 0.999 |
| Cirrhosis | 345 (3.1%) | 80 (7.6%) | **<0.001** |
| Chronic heart failure | 660 (6.0%) | 200 (18.9%) | **<0.001** |
| **Risk/triggering conditions** |  |  |  |
| Solid organ transplant | 90 (0.8%) | 25 (2.4%) | **0.028** |
| Hematopoietic stem cell transplant | 130 (1.2%) | 20 (1.9%) | 0.38 |
| COVID-19 | 185 (1.7%) | 50 (4.7%) | **<0.001** |
| Acute pancreatitis | 465 (4.2%) | 55 (5.2%) | 0.51 |
| Autoimmune conditions* | 1,475 (13.4%) | 190 (17.9%) | 0.066 |
| Cancer | 945 (8.6%) | 320 (30.2%) | **<0.001** |
| Solid organ tumor | 540 (5%) | 220 (21%) | **<0.001** |
| Hematological malignancies | 415 (4%) | 105 (10%) | **<0.001** |
| Pregnancy | 365 (3.3%) | <11 † | 0.022 |

* Autoimmune conditions were defined using ICD-10-CM codes and included systemic lupus erythematosus (SLE), Graves disease, immune thrombocytopenia (ITP), Addison’s disease, Sjögren syndrome, scleroderma, vitiligo, and acquired factor VIII inhibitor.

† Weighted count <11 to protect patient privacy

Abbreviations: chronic obstructive pulmonary disease (COPD); coronavirus disease of 2019 (COVID-19)

**Supplementary Table S4: In-Hospital Complications Stratified by Survival Status**

| **Variable** | **Survivors (Weighted N, %)** | **Non-survivors (Weighted N, %)** | **P - value** |
| --- | --- | --- | --- |
| Bacteremia | 140 (1.27%) | 10 (0.94%) | 0.681 |
| Sepsis | 1,210 (11.0%) | 475 (44.8%) | **<0.001** |
| AKI | 5,680 (51.5%) | 865 (81.6%) | **<0.001** |
| GI bleeding | 650 (5.89%) | 155 (14.6%) | **<0.001** |
| **Cardiac** |  |  |  |
| Acute coronary syndrome | 1,410 (12.8%) | 220 (20.8%) | **0.0013** |
| Arrhythmia | 1,060 (9.61%) | 305 (28.8%) | **<0.001** |
| Cardiac arrest | 65 (0.59%) | 255 (24.1%) | **<0.001** |
| Cardiogenic shock | 45 (0.41%) | 40 (3.77%) | **<0.001** |
| DVT/PE | 805 (7.30%) | 155 (14.6%) | **0.0002** |
| Acute heart failure | 410 (3.72%) | 140 (13.2%) | **<0.001** |
| **Neurologic** |  |  |  |
| Coma | 215 (1.95%) | 80 (7.55%) | **<0.001** |
| Seizure | 630 (5.71%) | 85 (8.02%) | 0.176 |
| Stroke (ischemic + hemorrhagic) | 1240 (7.8%) | 235 (15.9%) | **<0.001** |
| Acute ischemic stroke | 1,095 (9.93%) | 175 (16.5%) | **0.0031** |
| Intracranial hemorrhage (IPH) | 210 (1.90%) | 80 (7.55%) | **<0.001** |
| **Interventions** |  |  |  |
| TRALI | 25 (0.23%) | <11^ | 0.064 |
| RBC transfusion | 3,225 (29.2%) | 480 (45.3%) | **<0.001** |
| Platelet transfusion | 1,515 (13.7%) | 270 (25.5%) | **<0.001** |
| Mechanical ventilation | 1,335 (12.1%) | 700 (66.0%) | **<0.001** |
| Vasopressor use | 220 (2.00%) | 245 (23.1%) | **<0.001** |

Counts and percentages are **survey-weighted** (DISCWT). Column % are within survival status groups (Survivors total ≈ 11,030; non-survivors total ≈ 1,060). 
p-values are from **survey-adjusted chi-square tests** (design-based F).

**Supplementary Table S5. Multivariable Survey-Weighted Logistic Regression for Predictors of In-Hospital Mortality in TTP (Model A + B)**

| **Predictor** | **Model A OR (95% CI)** | **p-value** | **Model B OR (95% CI)** | **p-value** |
| --- | --- | --- | --- | --- |
| **Baseline characteristics** |  |  |  |  |
| Age (per year) | 1.02 (1.01–1.03) | **<0.001** | 1.01 (1.00–1.03) | **0.019** |
| Female sex | 1.15 (0.83–1.61) | 0.387 | 1.36 (0.97–1.92) | 0.078 |
| **Race (ref: White)** |  |  |  |  |
| Black | 0.54 (0.37–0.78) | **<0.001** | 0.60 (0.41–0.88) | **0.010** |
| Hispanic | 0.86 (0.50–1.47) | 0.589 | 0.98 (0.56–1.71) | 0.933 |
| Asian/Pacific Islander | 0.97 (0.42–2.26) | 0.95 | 1.06 (0.45–2.48) | 0.891 |
| Other race | 0.37 (0.09–1.45) | 0.156 | 0.41 (0.10–1.63) | 0.203 |
| **Exposure of interest** |  |  |  |  |
| Early TPE (≤1 day) | 0.49 (0.36–0.68) | **<0.001** | 0.66 (0.47–0.92) | **0.016** |
| **Comorbidities** |  |  |  |  |
| Chronic renal Failure | 1.13 (0.79-1.63) | 0.498 | 0.86 (0.59-1.25) | 0.419 |
| Chronic lung disease | 1.90 (1.29–2.77) | **<0.001** | 1.86 (1.22–2.84) | **0.004** |
| Chronic heart failure | 2.98 (1.85–4.53) | **<0.001** | 2.75 (1.70–4.45) | **<0.001** |
| COVID-19 infection | 4.30 (1.93–9.59) | **<0.001** | 2.90 (1.19–7.05) | **0.019** |
| Solid malignancy | 3.66 (2.33–5.73) | **<0.001** | 4.06 (2.53–6.53) | **<0.001** |
| Hematologic malignancy | 2.46 (1.38–4.39) | **0.002** | 2.28 (1.29–4.04) | **0.005** |
| Pregnancy | 0.33 (0.04-2.57) | 0.294 | 0.36 (0.05 – 2.80) | 0.328 |
| Solid organ transplant | 2.29 (0.70-7.08) | 0.175 | 2.43 (0.88-6.74) | 0.087 |
| **In-hospital complications (Model B only)** |  |  |  |  |
| Sepsis | — | — | 3.54 (2.49–5.03) | **<0.001** |
| Acute kidney injury | — | — | 2.49 (1.63–3.81) | **<0.001** |
| Acute myocardial infarction | — | — | 1.04 (0.65-1.65) | 0.869 |
| Acute heart failure | — | — | 1.99 (1.07–3.71) | **0.031** |
| Stroke (hemorrhagic + ischemic) | — | — | 1.94 (1.26–2.99) | **0.003** |
| GI bleeding | — | — | 1.97 (1.21–3.22) | **0.007** |

Model A adjusts for baseline demographic and clinical characteristics. Model B additionally adjusts for major in-hospital complications.

**Supplementary Table S6. Sensitivity Analysis Excluding Hospitalizations With Pregnancy, COVID-19 Infection, Malignancy, Transplant History, or Transplant Procedure During the Index Hospitalization**

| **Predictor** | **Model A OR (95% CI)** | **p-value** | **Model B OR (95% CI)** | **p-value** |
| --- | --- | --- | --- | --- |
| Age (per year) | 1.03 (1.01–1.04) | **<0.001** | 1.02 (1.00–1.03) | **0.01** |
| Female | 1.32 (0.89–1.97) | 0.167 | 1.54 (1.01–2.35) | **0.047** |
| Race (ref: White) |  |  |  |  |
| Black | 0.62 (0.40–0.94) | 0.024 | 0.71 (0.45–1.12) | 0.142 |
| Hispanic | 0.92 (0.50–1.68) | 0.781 | 1.08 (0.57–2.05) | 0.82 |
| Asian/Pacific Islander | 1.19 (0.46–3.06) | 0.72 | 1.41 (0.55–3.61) | 0.475 |
| Other race | 0.63 (0.16–2.52) | 0.513 | 0.84 (0.23–3.11) | 0.793 |
| Early TPE ≤1 day | 0.5 (0.34–0.73) | **<0.001** | 0.65 (0.44–0.97) | **0.036** |
| Renal failure | 1.48 (0.99–2.22) | 0.059 | 1.05 (0.67–1.62) | 0.839 |
| Chronic lung disease | 1.53 (0.96–2.45) | 0.075 | 1.44 (0.86–2.43) | 0.168 |
| Chronic heart failure | 3.01 (1.82–4.96) | **<0.001** | 2.81 (1.65–4.78) | **<0.001** |
| Sepsis | — | — | 3.58 (2.31–5.55) | **<0.001** |
| Acute kidney injury | — | — | 3.08 (1.78–5.33) | **<0.001** |
| Acute myocardial infarction | — | — | 1.19 (0.69–2.04) | 0.531 |
| Acute heart failure | — | — | 1.55 (0.74–3.25) | 0.249 |
| Neurologic complications | — | — | 1.8 (1.07-3.03) | **0.028** |
| Gastrointestinal bleeding | — | — | 1.64 (0.88-3.04) | 0.12 |

Transplant history or procedure was defined using ICD-10-CM/PCS codes indicating prior solid organ or hematopoietic stem cell transplant history, or solid organ/allogeneic hematopoietic stem cell transplantation during the index hospitalization.

**Supplementary Table S7. Sensitivity Analysis Using a Demographic Adjustment Model**

| **Predictor** | **OR** | **95% CI** | **p-value** |
| --- | --- | --- | --- |
| Early TPE ≤1 day | 0.42 | 0.31–0.57 | <0.001 |
| Age, per year | 1.03 | 1.02–1.04 | <0.001 |
| Female sex | 1.05 | 0.77–1.43 | 0.77 |
| Race (ref: White) |  |  |  |
| Black race | 0.5 | 0.35–0.72 | <0.001 |
| Hispanic ethnicity | 0.73 | 0.44–1.19 | 0.207 |
| Asian/Pacific Islander | 0.76 | 0.34–1.73 | 0.514 |
| Other race | 0.4 | 0.12–1.31 | 0.129 |

**Supplementary Figure S1**

**Figure legends:**

Supplementary Figure S1: Length of stay and total hospital charges stratified by early vs late initiation of plasma exchange.
